# A Vision Method for Detecting Citrus Separation Lines Using Line-Structured Light

**DOI:** 10.3390/jimaging11080265

**Published:** 2025-08-08

**Authors:** Qingcang Yu, Song Xue, Yang Zheng

**Affiliations:** School of Computer Science and Technology, Zhejiang Sci-Tech University, Hangzhou 310018, China; qcyu@zstu.edu.cn (Q.Y.); 2023220603108@mails.zstu.edu.cn (Y.Z.)

**Keywords:** line-structured light, 3D reconstruction, principal component analysis, skeleton extraction, least squares fitting

## Abstract

The detection of citrus separation lines is a crucial step in the citrus processing industry. Inspired by the achievements of line-structured light technology in surface defect detection, this paper proposes a method for detecting citrus separation lines based on line-structured light. Firstly, a gamma-corrected Otsu method is employed to extract the laser stripe region from the image. Secondly, an improved skeleton extraction algorithm is employed to mitigate the bifurcation errors inherent in original skeleton extraction algorithms while simultaneously acquiring 3D point cloud data of the citrus surface. Finally, the least squares progressive iterative approximation algorithm is applied to approximate the ideal surface curve; subsequently, principal component analysis is used to derive the normals of this ideally fitted curve. The deviation between each point (along its corresponding normal direction) and the actual geometric characteristic curve is then adopted as a quantitative index for separation lines positioning. The average similarity between the extracted separation lines and the manually defined standard separation lines reaches 92.5%. In total, 95% of the points on the separation lines obtained by this method have an error of less than 4 pixels. Experimental results demonstrate that through quantitative deviation analysis of geometric features, automatic detection and positioning of the separation lines are achieved, satisfying the requirements of high precision and non-destructiveness for automatic citrus splitting.

## 1. Introduction

Citrus, a globally cultivated fruit crop, holds significant importance in food processing, fresh produce markets, and related sectors [1]. The efficiency and quality of citrus processing directly impact the market competitiveness of products like canned segments and ready-to-eat items [2]. Accurate identification of citrus separation lines and optimized segmentation processes are crucial for enhancing product quality, reducing losses, and improving production efficiency [3]. Current citrus industry detection efforts remain limited to overall fruit recognition and detection [4], lacking specific focus on separation line identification [5]. Citrus processing enterprises mainly rely on manual visual inspection to identify separation lines. This experience-dependent method is often prone to errors, which cause segment damage and severely affect product quality and economic efficiency [6]. With growing consumer demands for superior appearance, integrity, and food safety of citrus products, the development of automated, non-destructive segmentation technologies has emerged as a critical breakthrough for industrial upgrading.

The spatial distribution characteristics of line-structured light fringes can reflect the changes in the surface morphology of objects [7]. This technology also has the advantages of strong environmental adaptability, high measurement accuracy, and a mature technical system. It is theoretically feasible and engineering valuable to achieve precise calculations of the spatial positions of separation lines by analyzing the deformation characteristics of laser fringe centerlines [8]. Dai et al. proposed a calibration method for linear structured light systems based on cubic spline interpolation functions, which is used to measure the 3D outer diameter contours of rotating workpieces during machining [9]. Chen et al. combined line-structured light with digital image correlation technology to realize 3D detection and visualization of cracks on human tooth surfaces [10]. Veinidis et al. developed a 3D reconstruction method for fish based on coded structured light, which is suitable for fusiform fish [11]. Song et al. designed a lightweight network, DcMcNet, to repair structured light stripe patterns of highly reflective objects, thereby improving the accuracy of 3D measurements [12]. The characteristics and limitations of the existing methods for citrus separation line detection are summarized in Table 1. While structured light research has made significant progress in technical methods [13,14,15,16], algorithm optimization, and expansion of application scenarios, bottlenecks remain in key technical links such as enhancing data acquisition accuracy.

**Figure 1:**
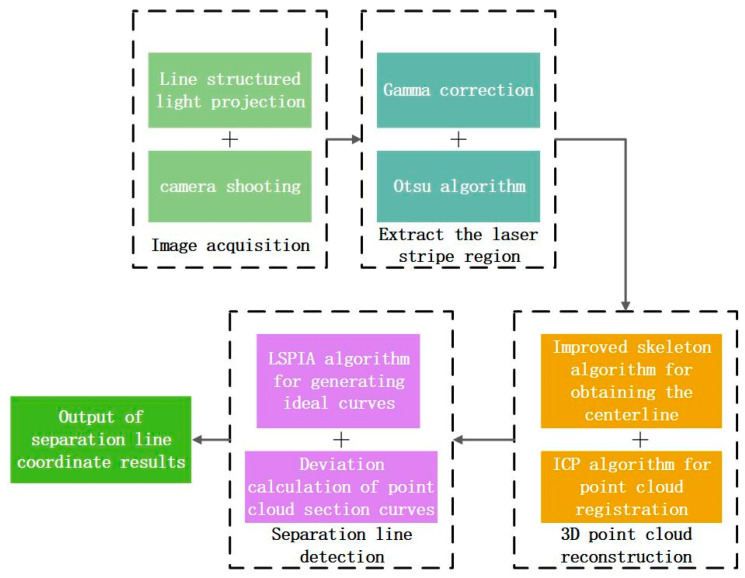
The overall flowchart of the experiment.

In this paper, we propose a novel method for 3D detection of citrus separation lines by leveraging the characteristic of line-structured light fringes that can reflect changes in the surface topography of objects. The overall flowchart of the experiment is illustrated in Figure 1. The innovation and significance of our research work are as follows:

1. To address interference from complex textures formed by the unique reticulated oil gland structure on citrus peels, we pioneeringly employ line-structured light for 3D reconstruction of citrus. Combined with PCA and LSPIA, the spatial locations of citrus separation lines are detected.
2. To improve the accuracy of the line-structured light 3D reconstruction system, the original skeleton extraction algorithm is improved to enhance the precision of extracting the centerline of laser fringes.

## 2. Measurement System Based on Line-Structured Light

### 2.1. Measuring Principle

**Figure 2:**
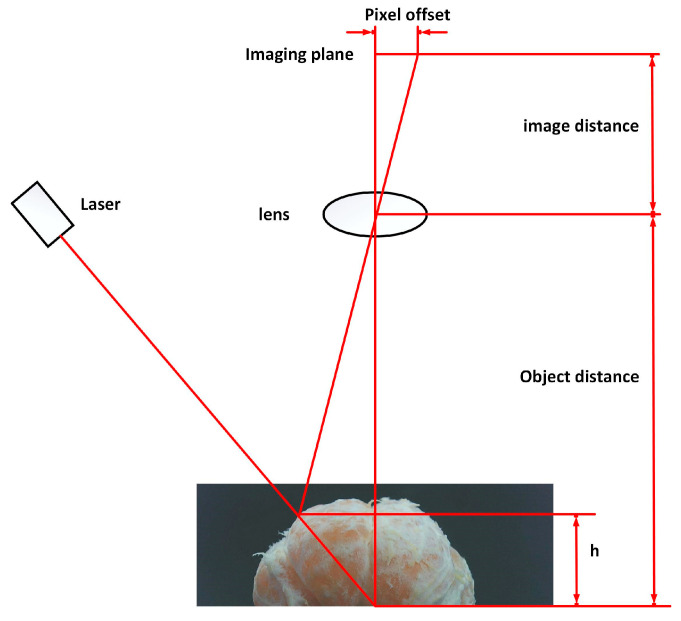
Schematic diagram of the laser triangulation method.

The principle of line-structured light imaging is shown in Figure 2, based on the geometric model of laser triangulation [17], where the plane of the line-structured light emitted by the laser forms a fixed triangulation angle θ with the camera optical axis. When the line-structured light is projected onto the surface of the citrus, the change in surface height results in a pixel shift in the laser streak in the camera imaging plane n. Setting the image distance (focal length) of the camera as $M_{1}$, and the object distance (distance from the reference plane to the lens) as $M_{2}$, the geometrical relationship is established by the principle of triangular similarity to obtain the citrus height h as:

(1) $$h=\frac{M_{2}n}{n+M_{1}tan\theta }$$

### 2.2. Experimental Platforms

**Figure 3:**
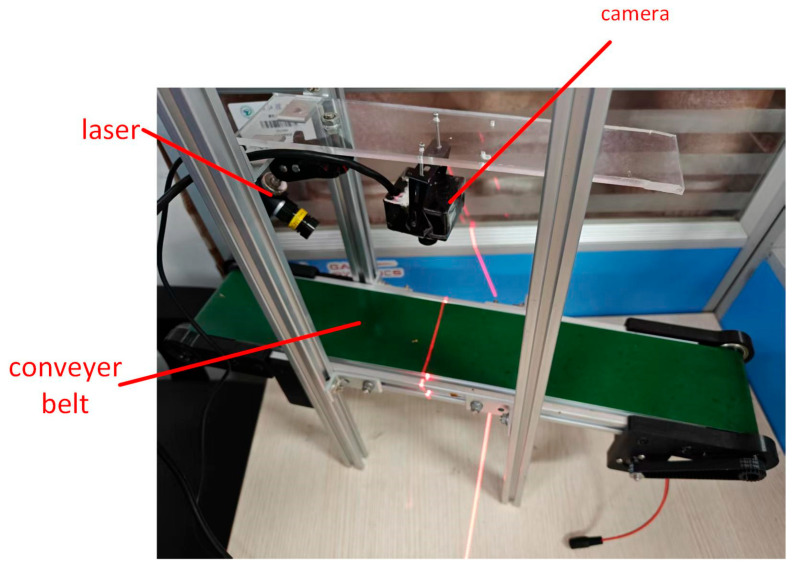
Experimental platforms.

Based on the imaging principle of line-structured light, a laser stripe acquisition system for citrus surfaces was constructed, as illustrated in Figure 3. The system comprises a RERVISION RER-USBGS1200P01 global shutter camera (resolution: 1920 × 1080 pixels; frame rate: 30 FPS) and a single-line green laser (wavelength: 520 nm). The manufacturer of this camera is RERVISION, which manufactures it in Shenzhen, Guangdong, China. The algorithm testing and development platform employs Python 3.8 and OpenCV 4.8, with the computer configured with an Intel Core i5-9300H processor and 16 GB of RAM. The manufacturer of Intel Core i5-9300H processor is Intel Corporation.

### 2.3. Camera Calibration

**Figure 4:**
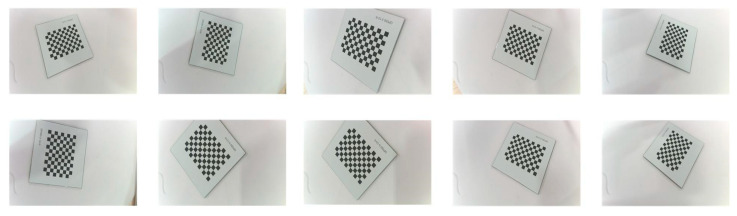
Calibration plate image.

In this paper, the camera’s intrinsic matrix, rotation vectors, translation vectors, and distortion coefficients were obtained using a calibration method based on a tessellated calibration plate [18]. The camera acquired ten calibration plate images with varying orientations, as shown in Figure 4. The calibration plate used was the GP025 high-precision black-and-white lattice plate, with specifications as follows: external dimensions of 25 mm × 25 mm; square lattice edge length of 1.5 mm; pattern array of 12 × 9; pattern size of 18 mm × 13.5 mm; and accuracy of ±0.005 mm. Calibration results are presented in Table 2.

## 3. Laser Centerline Extraction

### 3.1. Region of Interest Extraction

#### 3.1.1. Gamma Correction

Gamma correction [19] is an image enhancement technique based on a power–law relationship, primarily used to adjust the grayscale distribution of an image. It achieves this by nonlinearly mapping the image’s grayscale values using a specified gamma value, thereby enhancing the contrast of target regions. The gamma curve is defined by the formula:

(2) $$s\left(r\right)=cr^{\gamma }$$

Here, r represents the input grayscale value, s denotes the output grayscale value, c is a constant for adjusting overall brightness, and γ (gamma coefficient) determines the degree of nonlinearity in the mapping curve. In the Otsu algorithm incorporating gamma transformation, the gamma transform is primarily employed for preprocessing the input image. It adjusts the image’s grayscale distribution and enhances the contrast between the object and background, enabling the subsequent Otsu algorithm to more accurately determine the threshold that distinguishes the object from the background.

#### 3.1.2. OTSU Algorithm

The Otsu algorithm is an adaptive threshold segmentation method based on grayscale histograms [20]. Its core idea is to partition a grayscale image into two classes via thresholding, such that the discrepancy between these two classes is maximized. This discrepancy is quantified using inter-class variance: a larger variance indicates a higher degree of separability between the two classes and, consequently, a better segmentation result. For a grayscale image of size M×N with L gray levels, let $n_{i}$ denote the number of pixels corresponding to the gray level i, $p_{i}$ represent the probability of occurrence of pixels at a gray level i in the image, and $\mu_{G}$ denote the global grayscale mean. These quantities satisfy the following relationships:

(3) $$p_{i}=\frac{n_{i}}{MN}$$

(4) $$\mu_{G}=\sum_{i=0}^{L-1}ip_{i}$$

A threshold T is selected to partition the grayscale image into two classes: the background, consisting of pixels with grayscale values ⩽T, and the foreground, consisting of pixels with grayscale values >T. Let $w_{0}$ denote the pixel proportion of the background, $w_{1}$ the pixel proportion of the foreground, $\mu_{0}$ the mean grayscale of the background, and $\mu_{1}$ the mean grayscale of the foreground. These quantities are defined as follows:

(5) $$\left\{\begin{array}{c} w_{0}=\sum_{i=0}^{T}p_{i}\\ w_{1}=\sum_{i=T+1}^{L-1}p_{i} \end{array}\right.$$

(6) $$\left\{\begin{array}{c} \mu_{0}=\sum_{i=0}^{T}\frac{ip_{i}}{w_{0}}\\ \mu_{1}=\sum_{i=T+1}^{L-1}\frac{ip_{i}}{w_{1}} \end{array}\right.$$

The interclass variance $\sigma_{B}^{2}$ is given by:

(7) $$\sigma_{B}^{2}=w_{0}{\left(\mu_{0}-\mu_{G}\right)}^{2}+w_{1}{\left(\mu_{1}-\mu_{G}\right)}^{2}$$

This formula is derived from the adaptive threshold segmentation theory proposed by Otsu in 1979 [20]. The objective of the Otsu algorithm is to identify the threshold that maximizes $\sigma_{B}^{2}$ by iterating through all possible thresholds, which is regarded as the optimal threshold for image segmentation.

#### 3.1.3. Region of Interest Extraction Results

**Figure 5:**
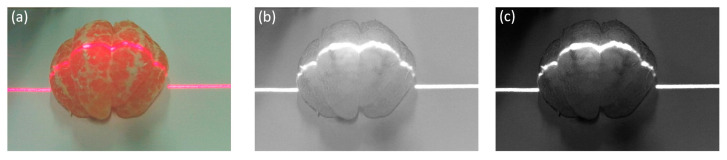
Image preprocessing results. (**a**) Original acquired image; (**b**) V-component image of the original image; (**c**) Gamma-corrected V-component image.

Gamma correction is applied to the V component extracted from the HSV color space of the original image, with a gamma coefficient set to 8, to suppress interference from reflective and bleed-through regions while enhancing the brightness of streak regions; this aims to maximize the interclass variance between the foreground and background. Figure 5 presents the results processed by our algorithm.

**Figure 6:**
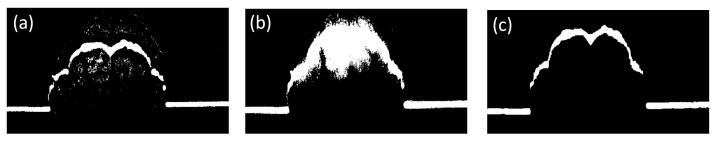
Region of interest extraction algorithm comparison image. (**a**) Adaptive thresholding algorithm; (**b**) Original Otsu algorithm; (**c**) Otsu algorithm based on gamma correction in this paper.

The Otsu algorithm performs well only for images with a clear foreground–background distinction, i.e., those with bimodal histograms. In our work, this algorithm is applied to extract the laser stripe region from the gamma-corrected V-component image. Figure 6 presents comparative results of laser stripe extraction using the adaptive thresholding algorithm [21], the original Otsu algorithm, and the Otsu algorithm based on gamma correction proposed in this paper. As shown in Figure 6, the adaptive thresholding algorithm can extract the main stripe region but introduces significant noise. The original Otsu algorithm classifies all brighter regions as foreground, resulting in the failure to extract most of the laser stripes. In contrast, our proposed algorithm effectively filters noise while preserving the stripe region with better connectivity, demonstrating strong adaptability to irregular surfaces.

### 3.2. Improved Skeleton Extraction Algorithm

The centerline of the laser is extracted by using the skeleton extraction algorithm. By iteratively eroding the binary image and stripping the boundary pixels until only the centerline remains [22], the region of interest in the image can be transformed into a single-pixel-wide skeleton while preserving the topological structure and geometric features of the laser.

Original skeleton extraction algorithms are prone to generating redundant branches. To accurately extract the laser centerline and eliminate these redundant branches, the improved skeleton extraction algorithm proposed in this paper is described as follows:

1. Let $M_{n}$ be the set of all pixel coordinates on the skeleton curve, and let max denote the length of the main branch of the skeleton, with an initial value of 0. The number of pixel points in $M_{n}$ is counted as n, and the number of other pixel points in the eight-neighborhood of pixel p is counted as $N_{p}$, with p = 1, 2, …, n;
2. Determine the point type of pixel p. If $N_{p}=1$, store pixel p in a new set of endpoints $M_{e}$; if $N_{p}>2$, store pixel p in a new set of branching points $M_{b}$;
3. Pixel i in the endpoint set $M_{e}$ adopts an eight-neighborhood tracking strategy, and when the next pixel t belonging to $M_{b}$ is accessed, the pixel is deposited into the access branch stack $V_{b}$;
4. Repeat step 3 until pixel j in the next set $M_{e}$ is found, note the path length $L_{ij}$ as the number of consecutive pixel points from pixel i to pixel j. Compare $L_{ij}$ with max, if $L_{ij}$ > max, the path from pixel i to pixel j is noted as the set of pixel coordinates R, max = $L_{ij}$;
5. Determine whether $V_{b}$ is empty, if non-empty, access the pixel back to the pixel at the top of the stack in $V_{b}$, and repeat steps 3, 4; if $V_{b}$ is an empty stack, output the set R of pixel coordinates of the main branch of the skeleton after the pruning process.

**Figure 7:**
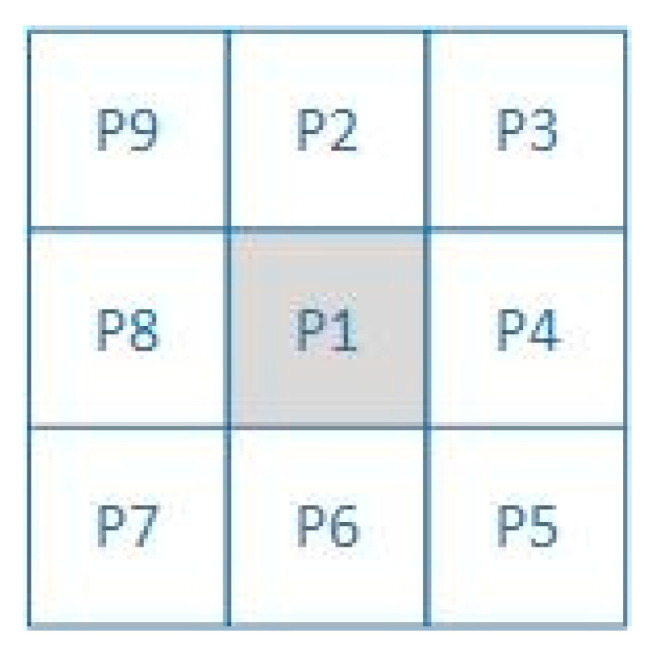
An 8-neighborhood range of pixel P1 (P2–P9 are neighborhood pixels).

**Figure 8:**
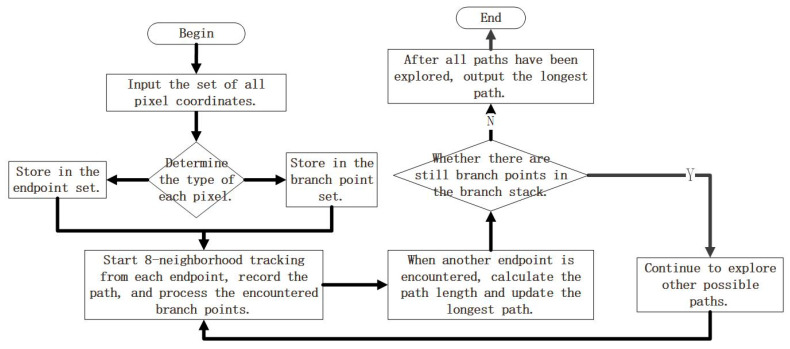
The steps of the improved skeleton extraction algorithm.

Here, the 8-neighborhood refers to the adjacent pixels around a pixel, as illustrated in Figure 7. The 8-neighborhood tracking strategy refers to searching for the next qualified pixel within the 8-neighborhood of a pixel. The steps of the improved skeleton extraction algorithm are shown in Figure 8. On an Intel Core i5-9300H processor, the average time cost for processing a single frame of a 1920 × 1080 pixel image is 0.8 s.

**Figure 9:**
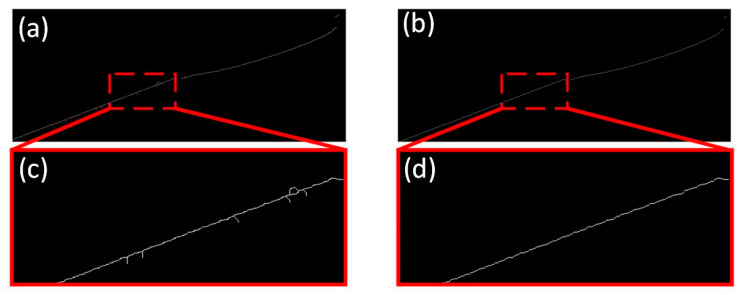
Comparison of results from the improved skeleton extraction algorithm. (**a**) shows the skeleton extracted by the original algorithm; (**b**) illustrates the skeleton extracted using the proposed algorithm; and (**c**,**d**) display the local enlargements of (**a**,**b**), respectively.

Figure 9 presents the results of the original skeleton extraction algorithm, the improved skeleton extraction algorithm, and their local enlargements. It is evident that our improved skeleton extraction algorithm achieves favorable performance with no redundant branches.

### 3.3. Laser Centerline Extraction Results

#### 3.3.1. Applicability Analysis

**Figure 10:**
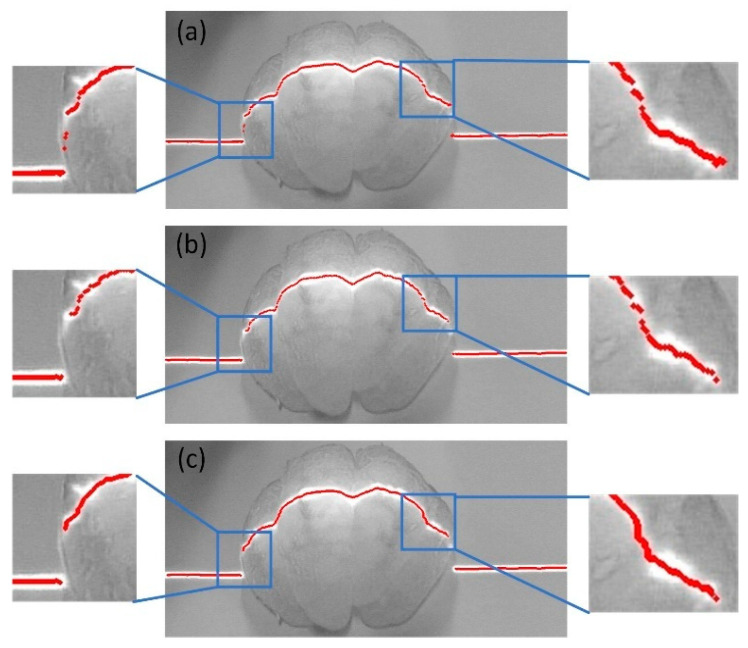
Comparison of stripe centerline extraction results. (**a**) Grayscale center of gravity method; (**b**) Steger algorithm; (**c**) Improved skeleton extraction algorithm.

Figure 10 presents the results of different laser stripe centerline extraction algorithms applied to the same stripe image. To verify the effectiveness of the geometric feature screening algorithm proposed in this paper for extracting the citrus separation line stripe, a comparative experiment was conducted with the grayscale centroid method and Steger’s algorithm. Specifically, two hundred citrus samples were selected, and the center lines of the laser stripe regions on the sample surfaces were extracted using each method.

From Figure 10, it can be observed that under conditions where the stripe curvature changes dramatically and the line width variations are complex:

- The Grayscale center of gravity method [23], which only considers the longitudinal grayscale distribution, exhibits a folding phenomenon. This results in significant deviations from the actual centerline, leading to poor extraction performance.
- The Steger algorithm [24] is susceptible to uneven illumination, causing the grayscale distribution to deviate from the Gaussian model and resulting in centerline discontinuities.
- In contrast, the proposed algorithm extracts a continuous centerline without breaks or deviations from the energy concentration region. Compared with the traditional methods, it achieves better smoothness and higher consistency with the laser stripe distribution.

#### 3.3.2. Accuracy Analysis

To evaluate the accuracy of laser stripe centerline extraction algorithms, this paper employs the root mean square error (RMSE) [25] as the accuracy metric. Specifically, the center points of the laser stripe extracted by different algorithms are first subjected to curve fitting. The RMSE, which quantifies the deviation of each center point from the fitted curve, is calculated as follows:

(8) $$R=\sqrt{\frac{\sum_{i=1}^{n}{\left(x_{i}-\bar{x}\right)}^{2}}{n}}$$

where n denotes the number of center points, $x_{i}$ represents the distance from the i-th center point to the fitted curve, and $\bar{x}$ is the mean distance. As shown in Table 3, we counted the RMSE of different algorithms on the same sample.

## 4. Method for Detecting the Separation Lines of Citrus

### 4.1. Point Cloud Registration

First, based on the structured light imaging principle described above, the pixel coordinates of the structured light stripe centerline extracted from a single frame of structured light stripe images are converted from the pixel coordinate system to the 3D spatial coordinate system using camera calibration parameters, generating single-frame 3D point cloud data.

In the experiment, 90 frames of structured light stripe images were collected at a conveyor speed of 20 mm/s. For each frame, the structured light stripe centerline was extracted, and 3D coordinate conversion was performed simultaneously, forming a sequence of 3D point cloud maps. The Iterative Closest Point (ICP) algorithm [26] was employed for frame-by-frame alignment of the sequential point cloud data. This algorithm achieves accurate spatial alignment of two adjacent point cloud frames by establishing correspondences between point sets and minimizing the sum of squared Euclidean distances between them.

**Figure 11:**
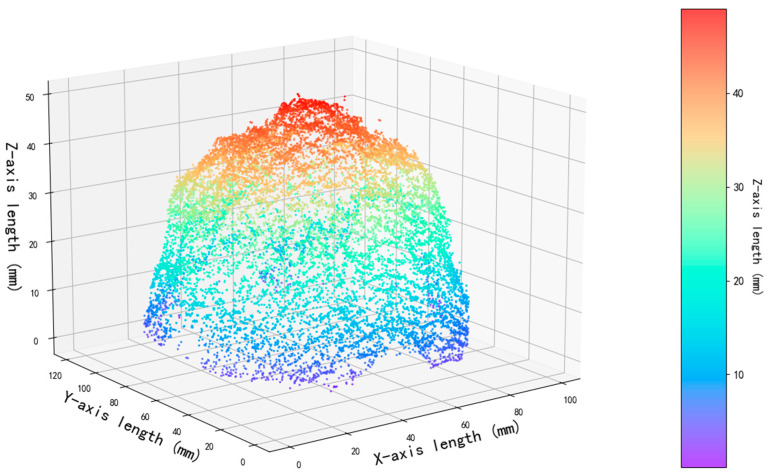
Three-dimensional reconstruction of a citrus.

Through a sequential integration strategy, the aligned sequential point clouds undergo global optimization, ultimately forming a globally consistent and complete 3D point cloud dataset—i.e., the surface point cloud data of the target sample (as shown in Figure 11).

### 4.2. Citrus Surface Curve Fitting

The least squares progressive iterative approximation (LSPIA) algorithm is employed to fit the ideal curves on the citrus surface. The ideal curve refers to the curve of the citrus section assuming that there is no separation line. Let $Q_{j}$ (*j* = 0, 1, …, *m*) denote the set of ordered data points to be fitted along the stripe centerline, with their corresponding parameters $u_{j}$ satisfying the following condition:

(9) $$u_{0}<u_{1}<\cdots <u_{m}$$

First, $P_{i}$, i = 0, 1, …, *n*, are selected as the control vertices of the cubic B-spline curve from the point set $Q_{j}$, and the initial fitting curve is defined.

(10) $$C^{\left(0\right)}\left(u\right)=\sum_{i=0}^{n}P_{i}^{\left(0\right)}N_{i}^{3}\left(u\right)$$

In Equation (10), $N_{i}^{3}\left(u\right)$ is the cubic B-spline basis function. $C^{\left(k\right)}\left(u\right)$ is the cubic B-spline fitting curve after k iterations; then the k+1 fitting curve is

(11) $$C^{\left(k+1\right)}\left(u\right)=\sum_{i=0}^{n}P_{i}^{\left(k+1\right)}N_{i}^{3}\left(u\right)$$

In Equation (11):

(12) $$\left\{\begin{array}{c} P_{i}^{\left(k+1\right)}=P_{i}^{\left(k\right)}+\delta_{i}^{\left(k\right)},i=0,1,\cdots ,n\\ \delta_{i}^{\left(k\right)}=\mu \sum_{j=0}^{m}N_{i}^{3}\left(u_{j}\right)r_{j}^{\left(k\right)},i=0,1,\cdots ,n\\ r_{j}^{\left(k\right)}=Q_{j}-C^{\left(k\right)}\left(u_{j}\right),j=0,1,\cdots ,n \end{array}\right.$$

μ is the step size parameter. Continue the iteration until the amount of control vertex change satisfies the given accuracy. When k = 0, 1, 2, …, *n*, Equations (13) and (14) can be obtained.

(13) $$P^{\left(k\right)}={\left[P_{0}^{\left(k\right)}P_{1}^{\left(k\right)}\cdots P_{n}^{\left(k\right)}\right]}^{T}$$

(14) $$Q^{\left(k\right)}={\left[Q_{0}Q_{1}\cdots Q_{m}\right]}^{T}$$

Then the algebraic expression for the control vertex computed by the LSPIA method is given by

(15) $$P^{\left(k+1\right)}=\left(I_{n+1}-\mu B^{T}B\right)P^{\left(k\right)}+\mu B^{T}Q=DP^{\left(k\right)}+c$$

In Equation (15), $c=\mu B^{T}Q$, $B={\left[N_{i}^{3}\left(u_{j}\right)\right]}_{j=0,i=0}^{m,n}$ is the configuration matrix, $I_{n+1}$ is the unit matrix of order $\left(n+1\right)$, and $D=I_{n+1}-\mu B^{T}B$ is the iteration matrix. The relevant iterative formulas are based on the LSPIA framework proposed by Deng and Lin [27]. The curve sequence generated by the LSPIA algorithm fitted by the cubic B-spline curve converges to the least squares fitting curve of the data point $Q_{j}$ to be fitted. If

(16) $$\mu =\frac{2}{\lambda_{0}+\lambda_{n}}$$

In (16), $\lambda_{0}$ and $\lambda_{n}$ are the maximum and minimum eigenvalues of $B^{T}B$, respectively. Then the method has the fastest convergence speed. At this time, the spectral radius of the iterative matrix *D* is

(17) $$\rho =\rho \left(D\right)=\frac{\lambda_{0}-\lambda_{n}}{\lambda_{0}+\lambda_{n}}$$

### 4.3. Separation Lines Detection Method

The gradient variation in the laser stripe is small along its extension direction but most significant in the normal direction. Given the limited variation in the normal direction of each pixel within a local region, the principal component analysis (PCA) method can be employed to construct a covariance matrix of gradient vectors in the stripe’s local region, followed by eigenvalue decomposition [28]. The eigenvector corresponding to the maximum eigenvalue is then taken as the normal direction of the curve in this region.

PCA is used to calculate the normal direction of the 3D point cloud cross-section curve, with the steps as follows: n two-dimensional data points $\left(x_{1},x_{2},\cdots ,x_{n}\right)$ are arranged into a 2×n matrix. The first step is to decentralize (de-mean) these two-dimensional data.

(18) $$x_{i}^{\prime }=x_{i}-\frac{1}{n}\sum_{i=1}^{n}x_{i}$$

The covariance matrix C is calculated as:

(19) $$C=\frac{1}{n-1}\sum_{i=1}^{n}x_{i}^{\prime }{x_{i}^{\prime }}^{T}=\frac{1}{n-1}XX^{T}$$

**Figure 12:**
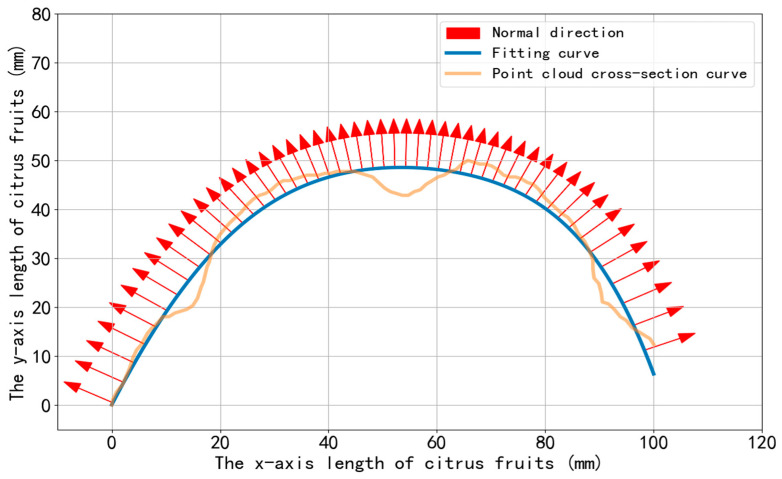
The ideal curve fitted to the citrus surface, along with the normal vectors of each point on it, and the cross-sectional curve of the citrus point cloud.

**Figure 13:**
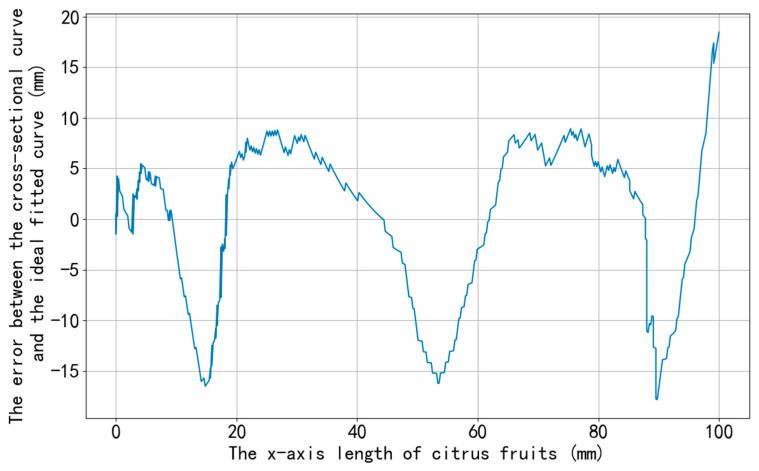
The deviation curve of the real segment line and the segment line extracted by our algorithm in the normal direction.

The eigenvalues and their corresponding eigenvectors of the covariance matrix C are computed. The calculation method of the covariance matrix refers to the research on structured light fringe normal extraction by Hu and Fang [28]. The normal direction of the stripe is defined as the eigenvector corresponding to the eigenvalue with the largest absolute value. As illustrated in Figure 12, for the ideal curve of the citrus surface, a statistical analysis is performed on the deviation between each point (along its normal direction) and the actual geometric characteristic curve. By setting a reasonable deviation threshold, the measurement data across the entire citrus surface is traversed and evaluated. As shown in Figure 13, if the deviation value of a point exceeds the preset threshold, the region is determined to contain a separation line feature. This method enables automatic detection and localization of the separation line through quantitative deviation analysis of geometric features.

**Figure 14:**
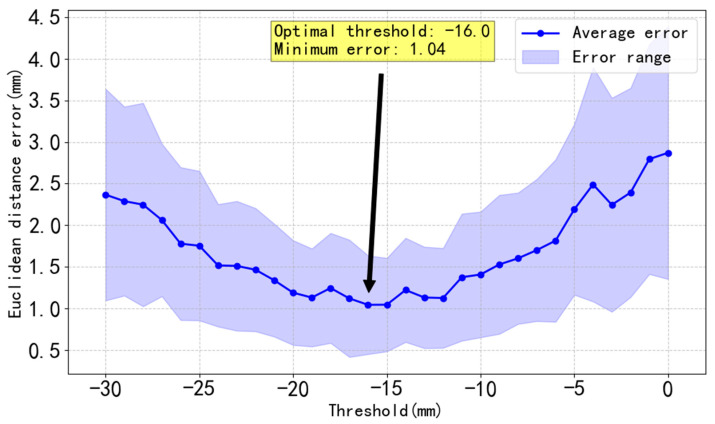
Image of errors between real separation lines and predicted separation lines as the threshold changes.

To find an appropriate deviation threshold for separation line detection, the Euclidean distance between each pixel coordinate of the predicted separation line and that of the real separation line is calculated, and the average error under the corresponding threshold is obtained, as shown in Figure 14.

### 4.4. Separation Line Detection Results

**Figure 15:**
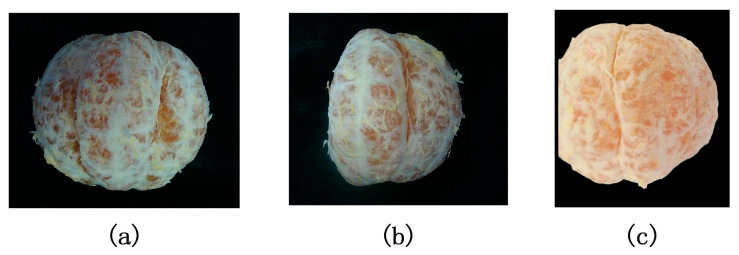
The citrus used in the experiment. (**a**) Rough surface with complex texture, (**b**) Incomplete sample, (**c**) Severe surface reflection.

To verify the effectiveness of the proposed method, citrus samples in different states were selected for experiments, as shown in Figure 15: (a) is a sample with complex texture interference on the surface, (b) is an incomplete sample, and (c) is a sample with strong reflection.

To further quantitatively evaluate the accuracy of the extracted separation lines, the similarity between the separation lines extracted by our method and those actually observed is calculated using the following formula:

(20) $$S=\frac{\sum \left(M_{i}\times N_{i}\right)}{\sqrt{\sum M_{i}^{2}\times \sum N_{i}^{2}}}$$

Here, $M_{i}$ represents the positions of separation lines manually extracted from images, while $N_{i}$ denotes the positions of separation lines extracted by our method and projected onto the xy plane.

**Figure 16:**
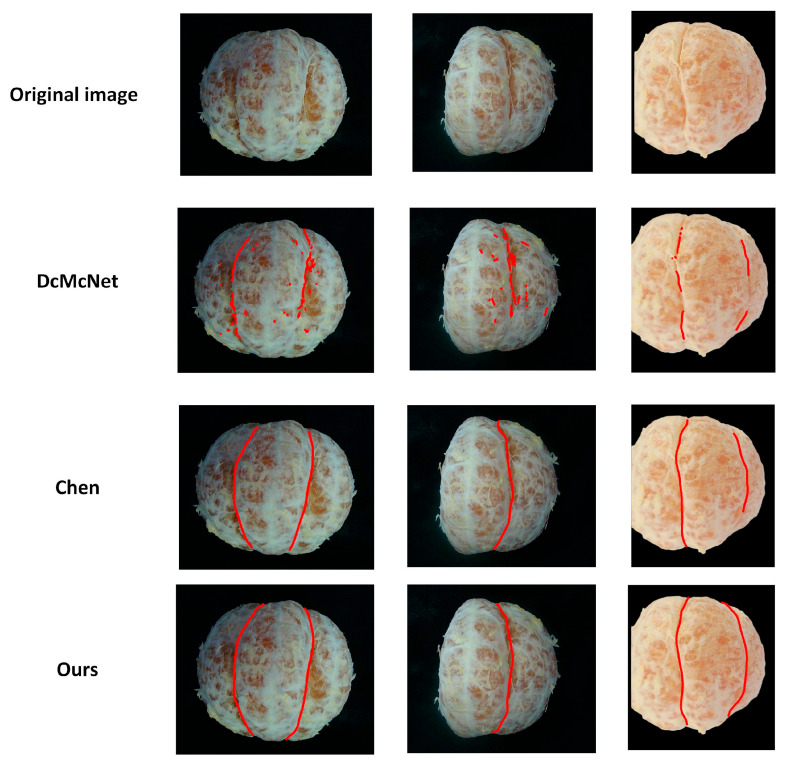
Results of citrus separation line detection. From top to bottom are the original image, DcMcNet, Chen’s method, and our method.

We conducted comparative experiments with DcMcNet and Chen [10] on citrus samples, and the results are presented in Table 4. From Figure 16, It can be observed that the citrus separation lines extracted by our method highly match the shape of those actually observed through visual inspection.

**Figure 17:**
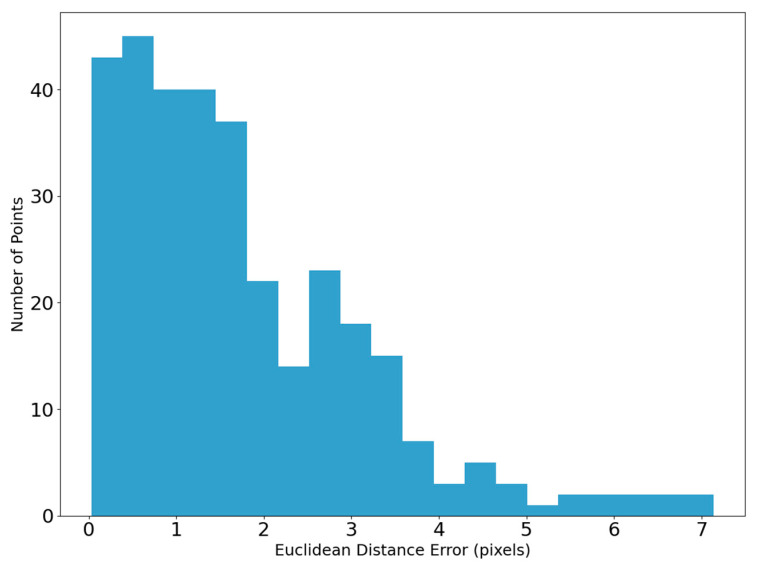
The error distribution between the real separation lines and the separation lines extracted by our method.

The error analysis was performed between the separation lines extracted by our method and the reference lines manually drawn along the separation lines using food-grade ink. Specifically, the points on the curves were matched one-to-one, and the Euclidean Distance Error between them was calculated. The curve errors are presented in Table 5. After multiple sample experiments, the average Mean Absolute Error (MAE) between the separation lines detected by our method and those manually marked is 1.809 mm, with an average RMSE of 2.252 mm. The error distribution frequency is shown in Figure 17, where 95% of the points on the separation lines obtained by this method have an error of less than 4 pixels.

Despite the presence of complex textures on the citrus surface and residual segment membranes from peeling—both of which cause diverse optical scattering and geometric interference—the proposed algorithm can still achieve accurate feature recognition when extracting separation lines from images captured under varying lighting conditions and texture backgrounds.

## 5. Discussion

Existing structured light technology has demonstrated high-precision advantages in surface defect detection. As reported in [10], it has been applied to tooth crack detection. However, when detecting separation lines on curved citrus surfaces with high reflectivity and complex textures, challenges such as susceptibility to interference in stripe center extraction and difficulties in quantitative detection of segment lines still remain. In this study, by improving the skeleton extraction algorithm and analyzing the deviation of citrus cross-sectional curves, structured light technology is effectively applied to citrus separation line detection for the first time, expanding its application scenarios in the field of precision processing of agricultural products.

As shown in Table 3, with RMSE as the evaluation metric, the improved skeleton extraction algorithm proposed in this paper achieves an average accuracy of 1.684 pixels. This represents a 41.8% improvement over the Grayscale center of gravity method and a 25.2% improvement compared to the Steger algorithm. The reason lies in that the improved skeleton extraction algorithm eliminates the redundant branch errors of original skeleton extraction algorithms through endpoint and branch point recognition as well as main branch tracking. Combined with a sub-pixel level extraction strategy, it significantly enhances the center positioning accuracy in scenarios with strong interference. This result lays a foundation for the subsequent high-precision 3D reconstruction of citrus.

**Figure 18:**
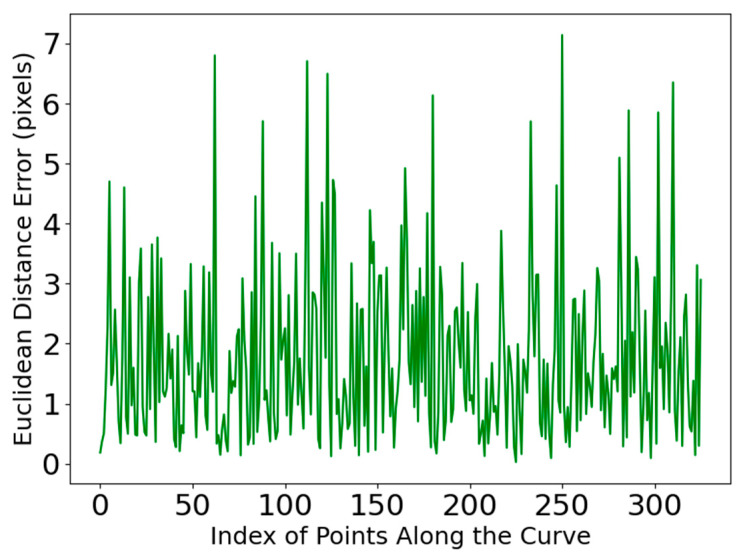
The error distribution along the coordinate indices of the separation lines extracted by our method.

As shown in Table 4, DcMcNet fails to accurately detect the position of the separation lines when disturbed by the complex textures on the citrus surface and when there are segment membranes in the gaps of the separation lines, with an average similarity of only 76%. Chen [10], which relies on planar vision, is affected by residual surface reflections and occlusions, resulting in an average similarity of 84.6%. The proposed method in this paper locates the separation line based on the surface deformation features of line-structured light and is not affected by illumination or grayscale. It achieves an average similarity of 92.5%, which is 7.9% higher than that of Chen [10] and 16.5% higher than that of DcMcNet, and has the highest consistency with the manually annotated standard. The curve of error variation along the path is displayed in Figure 18, indicating that the errors of the separation lines extracted by our method are relatively uniform, with no significant deviation in individual data points.

From the perspective of upgrading needs in the citrus processing industry, the proposed method, with an average similarity of 92.5% and sub-pixel level errors, can meet the high-precision requirements for separation line positioning in automated cutting equipment. It can effectively reduce pulp damage and juice loss caused by cutting deviations, providing key technical support for the transformation of citrus processing from manual to automated operations. This aligns with the development trend of non-destructive and high-efficiency detection in the food processing field.

The core value of this method is as follows: it solves the problems of low efficiency and poor accuracy in traditional manual detection, meeting the requirements for speed and precision in automated production lines; its non-contact design avoids fruit damage, which complies with hygiene standards in food processing. Therefore, this method has significant application prospects in scenarios such as citrus can processing and fresh-cut fruit production. Meanwhile, it also provides a reference for the 3D detection of subtle features of other curved-surface objects, such as micro-grooves and seam lines.

The limitation of this method is that it has currently only been validated on common citrus varieties. For pomelos with extremely rough peels or small lemons, their detection accuracy still requires improvement. Since our method relies on laser deformation, it performs poorly when the separation lines are occluded by obstacles. Additionally, the sample size needs to be further expanded to enhance the method’s generalization ability.

Future research can be optimized in two aspects: first, to address the issue of insufficient detection adaptability caused by differences among citrus varieties, deep learning will be introduced to assist in separation line detection; second, the computational processes of point cloud registration and curve fitting will be simplified to improve the algorithm’s real-time performance, thus meeting the requirements of high-speed detection in production lines.

## 6. Conclusions

To address the challenge of detecting separation lines under the interference of citrus surface reflections, wrinkles, and complex textures, while meeting the requirements of non-contact measurement to avoid damage to the pulp caused by mechanical contact, this paper proposes a citrus separation line detection method based on line-structured light. This method achieves accurate identification of the position of separation lines based on laser stripe deformation. The main conclusions are as follows:

- To address the issue where original skeleton extraction algorithms tend to generate redundant branches in complex textures, an improved skeleton extraction algorithm is proposed. The proposed algorithm achieves an average accuracy of 1.684 pixels, representing a 41.8% improvement over the Grayscale center of gravity method and a 25.2% improvement over the Steger algorithm. It significantly enhances centerline extraction accuracy and robustness in strongly interfering scenarios.
- The proposed method realizes automatic detection and an average similarity of 92.5% to manually defined standard separation lines. This meets the high-precision and non-destructive requirements of automated citrus splitting, offering technical support for improving processing efficiency, reducing product loss, and promoting industrial upgrading in the citrus processing sector.

## Figures and Tables

**Table 1:** Comparison of existing separation line detection methods.

Types of Methods	Advantages	Limitations
Machine vision	High computational efficiency	Susceptible to texture interference with low precision
Structured light technology	Enables 3D detection	Highly affected by surface reflection, with inaccurate detection in occluded areas
Deep learning	Strong anti-interference ability	Requires massive, labeled data with poor real-time performance

**Table 2:** Camera calibration parameters.

Parameters	Calibration Results
Camera Internal Parameters	$\left[\begin{array}{ccc} 4891.0 & 0 & 1333.5\\ 0 & 4109.9 & 613.5\\ 0 & 0 & 1 \end{array}\right]$
Radial Distortion Factor	$\left[\begin{array}{ccc} -222.8795 & 212.6786 & 268.8869 \end{array}\right]$
Tangential Distortion Factor	$\left[\begin{array}{cc} -0.3769 & -0.6711 \end{array}\right]$
Rotating Vector	$\left[\begin{array}{ccc} -0.0521 & -1.5855 & 0.0929 \end{array}\right]$
Translating Vector	$\left[\begin{array}{ccc} -1.0852 & 2.5743 & 159.7867 \end{array}\right]$

**Table 3:** Error analysis of laser center coordinates on the surface of citrus fruits.

Algorithms	RMSE						$\bar{R}$
	1	2	3	4	5	6	
Grayscale center of gravity method	3.860	4.071	1.737	1.215	3.073	3.414	2.895
Steger	3.388	2.843	1.786	1.218	2.557	1.726	2.253
Our algorithm	2.235	2.214	1.346	1.397	1.587	1.325	1.684

**Table 4:** Comparison of experimental results among different methods.

**M** **ethods**	**Similarity of Samples**			**Average**
	**1**	**2**	**3**	
DcMcNet	0.768	0.717	0.795	0.760
Chen [10]	0.824	0.837	0.878	0.846
Ours	0.923	0.916	0.937	0.925

**Table 5:** Error analysis of citrus separation line.

**Sample Number**	**MAE**	**RMSE**
1	1.853	2.278
2	1.795	2.173
3	1.726	2.141
4	1.718	2.131
5	1.860	2.352
6	1.884	2.392
7	1.828	2.298
average	1.809	2.252

Unit: mm.

## Data Availability

The data that support the findings of this study are available on request from the corresponding author (S.X.), upon reasonable request.

## Abbreviations

The following abbreviations are used in this manuscript:

RMSE	Root Mean Square Error
ICP	Iterative Closest Point
LSPIA	Least Squares Progressive Iterative Approximation
PCA	Principal Component Analysis
MAE	Mean Absolute Error
